# Mortality and its predictors among antiretroviral therapy naïve HIV-infected individuals with CD4 cell count ≥350 cells/mm^3^ compared to the general population: data from a population-based prospective HIV cohort in Uganda

**DOI:** 10.3402/gha.v7.21843

**Published:** 2014-01-15

**Authors:** Ben Masiira, Kathy Baisley, Billy N. Mayanja, Patrick Kazooba, Dermot Maher, Pontiano Kaleebu

**Affiliations:** 1 MRC/UVRI Uganda Research Unit on AIDS, Entebbe, Uganda; 2 Tropical Epidemiology Group, Department of Infectious Disease Epidemiology, London School of Hygiene and Tropical Medicine, London, UK

**Keywords:** mortality, HIV-infected, antiretroviral therapy naïve, general population, CD4 cell count ≥350 cells per mm^3^, rural Uganda

## Abstract

**Background:**

Evidence exists that even at high CD4 counts, mortality among HIV-infected antiretroviral therapy (ART) naïve individuals is higher than that in the general population. However, many developing countries still initiate ART at CD4 ≤350 cells/mm^3^.

**Objective:**

To compare mortality among HIV-infected ART naïve individuals with CD4 counts ≥350 cells/mm^3^ with mortality in the general Ugandan population and to investigate risk factors for death.

**Design:**

Population-based prospective HIV cohort.

**Methods:**

The study population consisted of HIV-infected people in rural southwest Uganda. Patients were reviewed at the study clinic every 3 months. CD4 cell count was measured every 6 months. Rate ratios were estimated using Poisson regression. Indirect methods were used to calculate standardised mortality ratios (SMRs).

**Results:**

A total of 374 participants with CD4 ≥350 cells/mm^3^ were followed for 1,328 person-years (PY) over which 27 deaths occurred. Mortality rates (MRs) (per 1,000 PY) were 20.34 (95% CI: 13.95–29.66) among all participants and 16.43 (10.48–25.75) among participants aged 15–49 years. Mortality was higher in periods during which participants had CD4 350–499 cells/mm^3^ than during periods of CD4 ≥500 cells/mm^3^ although the difference was not statistically significant [adjusted rate ratio (aRR)=1.52; 95% CI: 0.71–3.25]. Compared to the general Ugandan population aged 15–49 years, MRs were 123% higher among participants with CD4 ≥500 cells/mm^3^ (SMR: 223%, 95% CI: 127–393%) and 146% higher among participants with CD4 350–499 cells/mm^3^ (246%, 117%–516). After adjusting for current age, mortality was associated with increasing WHO clinical stage (aRR comparing stage 3 or 4 and stage 1: 10.18, 95% CI: 3.82–27.15) and decreasing body mass index (BMI) (aRR comparing categories ≤17.4 Kg/m^2^ and ≥18.5 Kg/m^2^: 6.11, 2.30–16.20).

**Conclusion:**

HIV-infected ART naïve individuals with CD4 count ≥350 cells/mm^3^ had a higher mortality than the general population. After adjusting for age, the main predictors of mortality were WHO clinical stage and BMI.

Mortality among HIV-infected individuals is much higher than in the general population (1–3) with the risk of death increasing as the CD4 cell count declines (4). Antiretroviral therapy (ART) is currently the most effective intervention for reducing mortality among HIV-infected individuals. The impact of ART in reducing mortality among HIV-infected people has been reported in developed countries (5) and low-income countries (6). However, in many developing countries, ART is still initiated when there is evidence of a significantly high risk of death, usually evidenced by clinical deterioration or low CD4 cell count. Currently, CD4 cell count is the most commonly used marker of immunological deterioration and HIV disease progression (7). In 2010, the World Health Organization (WHO) advised that HIV-infected people should be initiated on ART when their CD4 cell count is ≤350 cells/mm^3^ (8). As a result Uganda responded by issuing new national ART treatment guidelines shifting the threshold for initiating ART from ≤250 (9) to ≤350 cells/mm^3^ (10).

Over the last decade, ART has become more readily available with some countries even achieving universal ART access (11). There is a debate among experts concerning whether HIV-infected people are initiated on ART too late (12). Evidence shows that delayed ART initiation is associated with poorer outcomes such as morbidity (13), and that starting ART at higher CD4 cell count reduces the risk of death (14). Besides the effect of ART on reducing mortality and morbidity, early initiation of ART has been reported to reduce HIV transmission by up to 56% (15).

By providing ART only to HIV-infected people with a low CD4 cell count, it indirectly implies that mortality among HIV-infected individuals whose immune status is still competent, as evidenced by higher CD4 count, is low and similar to that observed in the general population. However, a large observational study in Europe and North America of over 40,000 patients reported higher death rates among HIV-infected ART naive individuals with CD4 cell counts ≥350 cells/mm^3^ than among the general population (16).

Several studies have reported predictors of mortality among HIV-infected people but mainly among those with advanced immunosuppression and on ART. A retrospective ART cohort study in Ethiopia reported weight loss ≥10%, bedridden functional status at baseline, advanced WHO clinical stage and CD4 cell count ≤200 cells/mm^3^ as independent predictors of death (17). Another hospital-based ART follow-up study in Thailand reported anaemia and severe immunosuppression at baseline as predictors of early mortality, whereas CD4 increase from a baseline CD4 of <50 cells/mm^3^, persistent anaemia and virological response at 6 months were identified as predictors of long-term mortality (18). On the other hand, a longitudinal pre-ART study in Zimbabwe reported haemoglobin and HIV clinical staging (CDC classification) as strong independent predictors of mortality, but this study did not assess predictors of mortality stratified by CD4 cell count (19).

Although most HIV-associated deaths occur in sub-Saharan Africa, information on mortality and its predictors among ART naïve individuals with high CD4 cell counts is still scarce, yet this information is useful in informing policy on the most appropriate time to start ART. Data on mortality patterns among HIV-infected persons with high CD4 cell counts are essential before expanding access to ART among this group.

We conducted the current study among HIV-infected ART naïve individuals with CD4 cell count ≥350 cells/mm^3^. We estimated overall mortality, compared mortality in the CD4 cell count category 350–499 cells/mm^3^ with that in the ≥500 cells/mm^3^ category, and compared mortality among study participants with mortality in the general population in Uganda. We also investigated risk factors for death among HIV-infected ART naïve persons with CD4 cell count ≥350 cells/mm^3^.

## Methods

### Setting and study population

The Rural Clinical Cohort (RCC) is an open HIV cohort that followed a total of 696 HIV-positive participants between 1992 and 2011. It is located in rural southwest Uganda and was established to study the natural history of HIV infection. The study setting has a stable, homogeneous population with most people belonging to the Baganda tribe. The majority of people are Christians and half the population is below 15 years of age (20). The RCC is nested within a large general population cohort (GPC) that conducts annual serosurveys and census on 20,000 residents of 25 study villages (21–23). In 1990, the RCC recruited one third of the HIV prevalent cases identified in the 1989 baseline GPC serosurvey; thereafter, HIV incident cases identified in subsequent annual GPC serosurveys were recruited (24). Following ART availability in Uganda in 2004, HIV-positive cases that remained in the GPC were invited to be screened and were enrolled into RCC if they qualified to start ART.

### Study participants’ recruitment and follow-up

Trained field workers visited individuals eligible for enrolment, explained the nature of the study and invited them to the study clinic where they gave informed consent before enrolment. After enrolment, study participants attended scheduled visits every 3 months at the study clinic. Data collected included medical, demographic, sexual and behavioural history and WHO clinical stage. HIV infection was staged according to WHO clinical staging (25). The WHO clinical staging system was developed in 1990 to guide physicians, in resource-limited settings, to make decisions related to management of HIV/AIDS patients. According to the WHO clinical staging system, HIV disease progression is grouped into four stages, i.e. stages 1, 2, 3 and 4. Study participants attended interim visits whenever ill and they received free investigations and treatment according to national guidelines (26) or were referred to a nearby health facility about 20 km away, for in-patient management. In 2004, ART was introduced for all eligible HIV-infected individuals according to the existing National ART guidelines at the time (27). These guidelines described ART eligibility then as: CD4 count of 200 cells/mm^3^ or below, or WHO clinical stage 4, or advanced stage 3 with persistent or recurrent oral thrush and invasive bacterial infections regardless of CD4 count, or CD4 count of 250 cells/mm^3^ or below in pregnancy. Although the National Cotrimoxazole prophylaxis guidelines were released in 2005 (28), cotrimoxazole prophylaxis among HIV-infected individuals had been introduced in the cohort in 2004. To minimize loss to follow-up, study participants were transported to the study clinic (or given transport refund) for their scheduled visits, and those who missed their scheduled visits were visited at home within 3 days. Field workers who received information about a study participant's death by the deceased's next of kin, relative, friend or community-based recorders in the GPC notified study clinicians of deaths.

### Laboratory assessment

CD4 cell counts were measured every 6 months among HIV-infected study participants not yet on ART, using FACS Count Machine (Becton Dickinson, San Jose, California, USA). HIV viral load measurement was done at enrolment and annually thereafter, using two methods during the study. Up to September 2007, we used the VERSANT RNA 3.0 (Bayer, Bayer HealthCare, NY, USA) assay (lower detection limit=50 copies/ml). However, due to problems with lack of reagents and lack of service support, we used the COBAS Amplicor MONITOR 1.5 (Roche, Roche Molecular Systems, NJ, USA) assay (lower detection limit=400 copies/ml) from October 2007 onwards. Other laboratory tests done at scheduled visits included thick blood malaria smears, stool analysis, urine examination and full blood counts.

### Outcome

The outcome of interest was death, with mortality rates (MRs) estimated from deaths per 1,000 person-years (PY), and age- and sex- standardised MRs.

### Exposures

The primary exposure variable was current CD4 count 350–499 and ≥500 cells/mm^3^ among ART naïve HIV-infected individuals. The following secondary exposure variables were measured at enrolment: age, sex, tobacco consumption (measured as years of tobacco consumption and number of times tobacco is consumed each day), alcohol consumption (measured as number of units consumed and number of consumption days each week), level of education (none, primary and secondary or above) and height (in centimetres). The following secondary exposure variables were measured at enrolment and every 3 months: marital status (married, with steady sexual partner, single, divorced or widowed), diarrhoea (three or more loose motions each day) for more than 1 month, WHO clinical stage (measured as stage 1, 2, 3 or 4), malaria (diagnosed from blood smear), height, weight (in kilograms) and viral load.

### Statistical analysis

Data were double-entered, cleaned and validated using a MS Access database and analysed using STATA version 11.0 (StataCorp, College Station, Texas, USA). Mortality data from the general Ugandan population were extracted from the 2011 Uganda Demographic and Health Survey (DHS) (29). At the time of the DHS, there were a total of 80 districts from which a representative sample of 9,854 households was selected using multistage sampling. Age-specific MRs, for women and men aged 15–49 years, were calculated for the 7 years before the survey was carried out (mid-2004 to mid-2011).

Because our primary objective was to estimate mortality among HIV-positive persons who were not yet eligible for ART, person-time at risk started at the first CD4 cell count ≥350 cells/mm^3^ and was censored at the earliest of death, CD4 cell count <350 cells/mm^3^, start of ART or date last seen. Multiple episodes of observation and gaps were allowed if an individual's CD4 count fell below 350 and then later increased to ≥350 cells/mm^3^ without ART initiation. Loss to follow-up was defined as a participant who was not known to be dead but had not been seen at the study clinic for at least 1 year. If a participant missed a CD4 cell count at a scheduled visit, the value from the previous visit was carried forward, if within the last 6 months. CD4 cell counts were considered to be unknown during periods when there was >6 months since the last CD4 cell count and this period was excluded from the analysis. Similarly, viral load data were carried forward if the previous measurement was less than 12 months ago. Viral load measurements were considered to be unknown in the periods when there was >12 months since the last measurement.

Baseline characteristics (defined as the point when the participant first entered the analysis) were calculated and tabulated by CD4 count category. Calendar time was divided into two periods before ART was available (1992–1999 and 2000–2003) and two periods after ART was available (2004–2007 and 2008–2011). MRs were calculated using standard person-year methods. Crude and adjusted rate ratios (RR) were calculated using Poisson regression and associations assessed using likelihood ratio tests (LRT). Adjusted RRs (aRR) for CD4 count category, adjusted for the effect of current age, sex and calendar period as a priori confounders, were calculated. Effect modification of CD4 cell count category by age, sex and calendar period was also assessed.

To identify independent risk factors for mortality, a multivariate Poisson model was constructed. Current age was considered to be a priori confounder and included in all models. Variables that were measured at each visit were analysed as time-updated exposures. Risk factors that were associated with mortality at *P*<0.20 in the univariate analysis were assessed for inclusion in the multivariate model. A forward stepwise method was used, starting with the factor that had the strongest association and adding other factors to the model one by one; those that were associated at *P*<0.05 were retained. In the final model, effect modification between CD4 count category and each of the other covariates was assessed. Survival probabilities were evaluated using Kaplan-Meier curves stratified by CD4 count categories. Standardised mortality ratios (SMRs) for age and sex were calculated using the indirect method and the analysis was restricted to 15–49 years of age to match with the Uganda DHS data. With the indirect method of standardisation, the age- and sex-specific MRs in the Ugandan population are multiplied by the person-years in each sex and age group in our study cohort to obtain the expected number of deaths in our cohort if the two populations had the same mortality.

### Ethical considerations

The RCC from which this data was extracted received ethical approval from the Uganda Virus Research Institute Science and Ethics committee and the Uganda National Council for Science and Technology. All informed consent and confidentiality procedures were adhered to.

## Results

### Characteristics of participants

Between June 1992 and March 2011, 374 participants had a CD4 cell count ≥350 cells/mm^3^ on at least one occasion during follow-up (Table 1). Overall median age among study participants was 31 years (IQR: 25–41), and 55% (*n*=204) were females. At baseline, defined as entry to the analysis, 71% (*n*=264) of participants had a CD4 cell count ≥500 cells/mm^3^, and 29% had a CD4 cell count of 350–499 cells/mm^3^. The majority (*n*=243, 65%) of participants were married or had a steady sexual partner, and had some primary education (66%). Most participants (65%) reported not using tobacco and not consuming alcohol (69%). Sixteen percent of participants had a body mass index (BMI) <18.5 Kg/m^2^, 8% had haemoglobin level of <11 g/dL, and 3.2% were in WHO clinical stage 3 or 4. There were no significant differences in the distribution of the baseline characteristics by CD4 cell count category.

**Table 1:** Distribution of characteristics at study entry by CD4 cell count category

	CD4 cell count (cells/mm^3^) *N*=374		
		
Characteristic	350–499 *n*=110 (column %)	≥500 *n*=264 (column %)	Total (column %)
Age (years)		***P*= 0.49**	
14–19	9 (8.2)	22 (8.3)	31 (8.3)
20–29	33 (30.0)	103 (39.0)	136 (36.4)
30–39	33 (30.0)	70 (26.5)	103 (27.5)
40–49	22 (20.0)	39 (14.8)	61 (16.3)
≥50	13 (11.8)	30 (11.4)	43 (11.5)
Overall median age (IQR)	32 (25–43)	30 (24–40)	31 (25–41)
Sex		***P*= 0.11**	
Female	53 (48.2)	151 (57.2)	204 (54.5)
Male	57 (51.8)	113 (42.8)	170 (45.5)
Tribe		***P*= 0.12**	
Baganda	57 (51.8)	162 (61.4)	219 (58.6)
Banyarwanda	28 (25.5)	59 (22.3)	87 (23.3)
Other	17 (15.5)	36 (13.6)	53 (14.2)
Missing	8 (7.3)	7 (2.7)	15 (4.0)
Religion		***P*= 0.07**	
Catholic	61 (55.5)	170 (64.4)	231 (61.8)
Church of Uganda	24 (21.8)	37 (14.0)	61 (16.3)
Other	18 (16.4)	50 (18.9)	68 (18.2)
Missing	7 (6.4)	7 (2.7)	14 (3.7)
Marital status		***P*= 0.88**	
Married /Steady sexual partner	73 (66.4)	170 (64.4)	243 (65.0)
Single	9 (8.2)	25 (9.5)	34 (9.1)
Divorced/widowed	27 (24.5)	64 (24.2)	91 (24.3)
Missing	1 (0.9)	5 (1.9)	6 (1.6)
Education level		***P*= 0.47**	
None	14 (12.7)	43 (16.3)	57 (15.2)
Primary	72 (65.5)	175 (66.3)	247 (66.0)
Secondary and above	18 (16.4)	39 (14.8)	57 (15.2)
Missing	6 (5.5)	7 (2.7)	13 (3.5)
Alcohol consumption (times/week)		***P*= 0.14**	
Don't use	69 (62.7)	190 (72.0)	259 (69.3)
1–3	30 (27.3)	59 (22.4)	89 (23.8)
≥4	10 (9.1)	15 (5.7)	25 (6.7)
Missing	1 (0.9)	0	1 (0.3)
Years of tobacco use		***P*= 0.79**	
Don't use	70 (63.6)	172 (65.2)	242 (64.7)
≤5	10 (9.1)	31 (11.7)	41 (11.0)
6–10	7 (6.4)	14 (5.3)	21 (5.6)
11–19	12 (10.9)	21 (8.0)	33 (8.8)
≥20	4 (3.6)	14 (5.3)	18 (4.8)
Missing	7 (6.4)	12 (4.5)	19 (5.1)
Body mass index (Kg/m^2^)		***P*= 0.46**	
≥25.0	5 (4.5)	21 (8.0)	26 (7.0)
18.5–<25	83 (75.5)	201 (76.1)	284 (75.9)
<18.5	20 (18.2)	40 (15.2)	60 (16.0)
Missing	2 (1.8)	2 (0.8)	4 (1.1)
Haemoglobin (g/dL)		***P*= 0.70**	
<11.0	9 (8.2)	21 (8.0)	30 (8.0)
≥ 11.0	76 (69.1)	172 (65.2)	248 (66.3)
Missing	25 (22.7)	71 (26.9)	96 (25.7)
WHO stage		***P*= 0.64**	
1	91 (82.7)	215 (81.4)	306 (81.8)
2	13 (11.8)	29 (11.0)	42 (11.2)
3 and 4	4 (3.6)	8 (3.0)	12 (3.2)
Missing	2 (1.8)	12 (4.5)	14 (3.7)

*P-*value from Chi-squared test for comparison of proportions.

### Missing data

At baseline, only a small proportion of participants (<10%) had missing data for most covariates, except for haemoglobin that was missing for 23% participants (Table 1). During follow-up, CD4 counts were missing in 15% of the total observation time, and viral load measurements were missing during 31% of observation time. Other covariates were missing for <2% of observation time, except for haemoglobin which was missing for 9% of observation time.

### Follow-up time and survival among participants with CD4 ≥350 cells per mm^**3**^

Thirteen (3.5%) participants were censored due to loss to follow-up, of whom 11 (85%) had a CD4 cell count ≥500 cells/mm^3^ and two (15%) had a CD4 cell count 350–499 cells/mm^3^. Overall, the 374 participants in the periods with CD4 cell count ≥350 cells/mm^3^ contributed 1,328 PY at risk. There were 2,921 visits made by 295 participants during periods of CD4 count 350–499 cells/mm^3^; 60% (*n*=1,766) of visits were scheduled follow-up visits and the remainder were interim (unscheduled) visits. At 43 scheduled visits (2.4%), participants reported having been hospitalised since their last visit; overall, 34 participants (11.5%) were hospitalised at least once during a period when their CD4 count was 350–499 cells/mm^3^. There were 5,521 visits made by 309 participants during periods of CD4 counts ≥500 cells/mm^3^; 64% (*n*=3,551) of visits were scheduled follow-up visits. Participants reported having been hospitalised at 66 scheduled visits (1.9%); overall, 48 participants (15.5%) were hospitalised at least once when their CD4 count was ≥500 cells/mm^3^.

There were 27 deaths among the study participants (overall MR=20.34/1,000 PY; 95% CI: 13.95–29.66). Twelve deaths occurred among individuals with CD4 cell count 350–499 (MR=27.10/1,000 PY; 95% CI: 15.37–47.66) and 15 deaths among individuals with CD4 count ≥500 (MR=17.00/1,000 PY; 95% CI: 10.23–28.14) (Table 4). Twelve deaths (MR=17.20/1,000 PY; 95% CI: 9.77–30.29) occurred in the pre-ART period (1990–2003) and 15 deaths (MR=23.80/1,000 PY; 95% CI: 14.35–39.48) occurred in the ART period (2004–2011) (Data not shown).

**Fig. 1:**
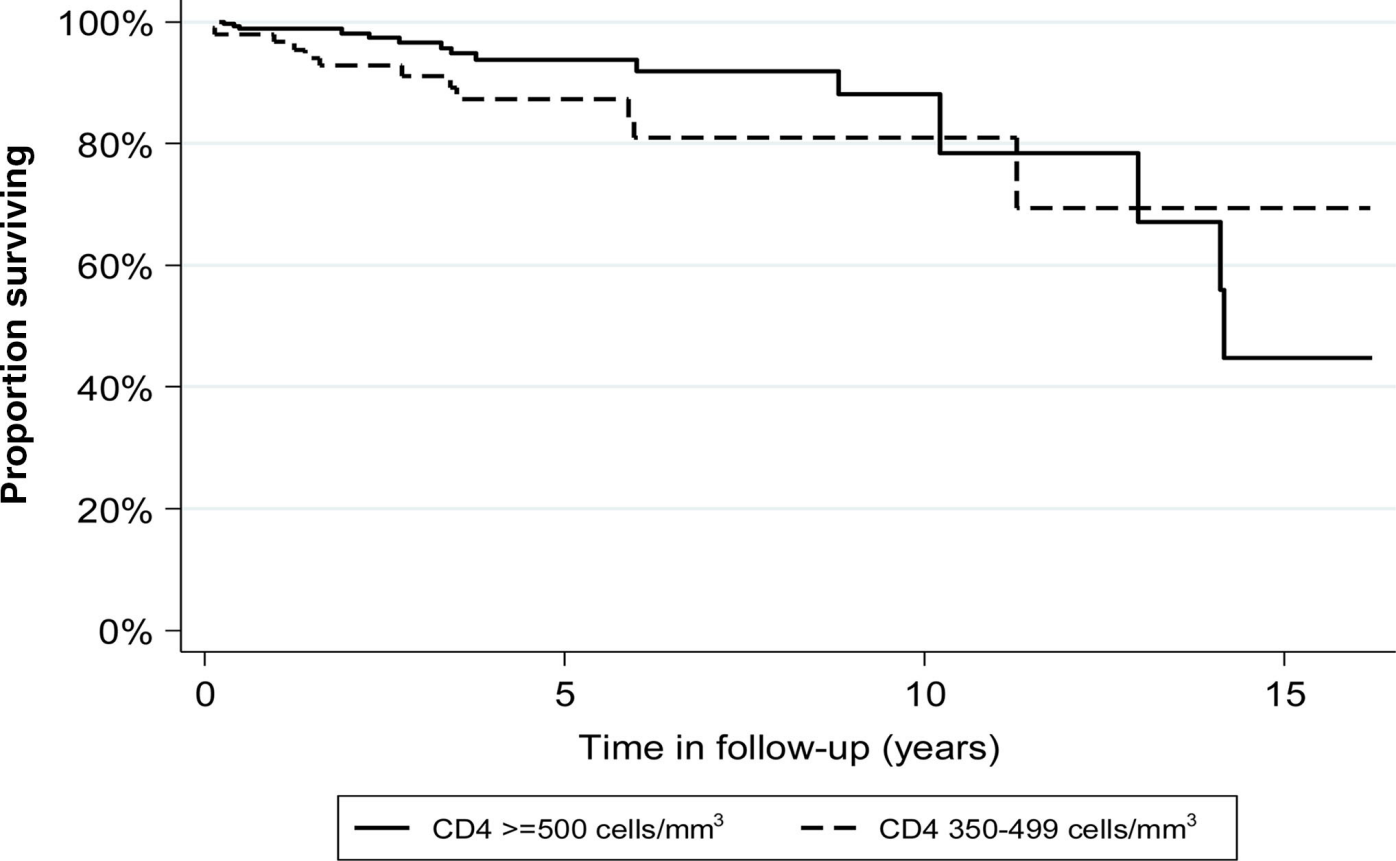
Kaplan-Meier survival function for cohort, by CD4 cell count category.^a^ ^a^ *P*-value for log-rank test for equality of survival function=0.14.

Although not statistically significant, the unadjusted MR in participants with CD4 cell count 350–499 cells/mm^3^ was 1.6 times higher than in those with CD4 cell count ≥500 cells/mm^3^ (Table 2 and Fig. 1). After adjusting for age, sex and calendar period, the risk of death among those with CD4 cell count 350–499 cells/mm^3^ remained higher than among those with CD4 cell count ≥500 cells/mm^3^, but the difference was still not statistically significant (aRR:1.44; 95% CI: 0.71–3.25) (Table 2). There was some evidence that the effect of CD4 cell count category varied by calendar period. Participants with CD4 cell count category 350–499 cells/mm^3^ had a higher MR than those with CD4 cell count ≥500 cells/mm^3^ in the period before 2004, however, after 2004, the MR in participants with CD4 cell count 350–499 cells/mm^3^ was lower than that that in participants with CD4 cell counts ≥500 cells/mm^3^ (*P*-value for interaction=0.02). There was no evidence of effect modification by age (*P*=0.38) or sex (*P*=0.97).

**Table 2:** Crude rate ratios and rate ratios adjusted for current age, sex and time period, by CD4 cell count category

CD4 category (cells/mm^3^)	Deaths (person-years)	Mortality rate/1,000 person-years (95% CI)	Crude rate ratio (95% CI)	Adjusted rate ratio (95% CI)
			*P*=0.23†	*P*=0.29†
≥500	15 (884)	17.00 (10.23–28.14)	1.00	1.00
350–499	12 (443)	27.07 (15.37–47.66)	1.60 (0.75–3.41)	1.52 (0.71–3.25)

95% CI=95% confidence interval.

† *P*-value from likelihood ratio test.

### Mortality in participants with CD4 ≥350 cells per mm^**3**^ compared with the general population

Among individuals aged 15–49 years, the MR in the Ugandan population was 5.66/1,000 PY (95% CI: 5.46–5.87), compared with 16.43/1000 PY (95% CI: 10.48–25.75) among study participants with CD4 count ≥350 cells/mm^3^. Mortality was 146% higher among study participants with CD4 counts of 350–499 cells/mm^3^ (SMR=246%; 95% CI: 117–516%) and 123% higher among study participants with CD4 counts of ≥500 cells/mm^3^ (SMR: 223%; 95% CI: 127–393%). Mortality in male participants with CD4 ≥350 cells/mm^3^ was more than double that in males in the general Ugandan population (SMR: 313%; 95% CI: 173–565%). Mortality in female participants with CD4 ≥350 cells/mm^3^ was over 100% higher than that in females in the general Ugandan population (SMR: 245%; 95% CI: 123–490%; Table 3).

**Table 3:** Standardised mortality ratios (SMRs), by sex, among individuals ≤49 years and CD4 cell count ≥350 cells/mm^3^

	Observed cohort deaths	Observed follow-up (person-years)	Mortality rate in standard population (per 1,000 person-years)	Expected deaths	SMR (95% CI)
Age (≥500 cells/mm^3^)					
15–29 years	1	325	3.5	1.14	
30–39 years	4	300	8.2	2.46	
40–49 years	7	150	11.9	1.79	
Overall	12	775	5.7	5.39	223% (127–393%)
Age (350–499 cells/mm^3^)					
15–29 years	1	119	3.5	0.42	
30–39 years	4	178	8.2	1.46	
40–49 years	2	81	11.9	0.96	
Overall	7	378	5.7	2.84	246% (117–516%)
Males					
≥500 cells/mm^3^	6	353		2.29	
350–499 cells/mm^3^	5	188		1.22	
Overall	11	541	6.5	3.51	313% (173–565)
Females					
≥500 cells/mm^3^	6	422		2.24	
350–499 cells/mm^3^	2	194		1.03	
Overall	8	616		3.27	245% (123–490%)

### Risk factors for mortality among participants with CD4 ≥350 cells/mm^**3**^

In the univariate analysis, mortality was strongly associated with increasing current age (RR=2.8; 95% CI: 1.1–7.5 comparing those aged ≥50 years with those 30–39 years; *P*=0.001) and decreasing BMI (RR=17.1; 95% CI: 7.0–41.8, comparing BMI <17.5 Kg/m^2^ with BMI ≥18.5 Kg/m^2^; *P*<0.001). Mortality was also strongly associated with a haemoglobin level <11 g/dL (RR=3.8; 95% CI=1.6–8.8; *P*=0.005) and increasing WHO clinical stage (17.9; 95% CI: 7.4–43.3, comparing WHO stage 3 or 4 and WHO stage 1; *P*<0.001). There was also an association between mortality and increasing viral load (RR=4.4; 95% CI: 1.1–18.5, comparing viral load categories ≥250,000 copies/ml and <25,000 copies/ml; *P*=0.003).

In the multivariate analysis, after adjusting for current age, independent factors associated with mortality were decreasing BMI (aRR=6.1; 95% CI: 2.3–16.2, comparing BMI <17.5 kg/m^2^ to BMI ≥18.5 kg/m^2^; *P*<0.001) and increasing WHO clinical stage (aRR=10.2, 95% CI: 3.8–27.2 comparing WHO stage 3 or 4 to WHO stage 1; *P*<0.001) (Table 4). After adjusting for current age, BMI and WHO stage, although mortality was slightly higher in those with CD4 cell count 350–499, there was no evidence of an association with CD4 count category (aRR=1.2, 95% CI; 0.6–2.7; *P*=0.63). There was no evidence of effect modification of CD4 count category by any of the covariates in the final model (*P*-value for interaction with age=0.42, with BMI=0.77 and with WHO stage=0.36).

**Table 4:** Univariate and multivariate analysis of factors associated with death among individuals with CD4 ≥350 cells/mm^3^

Variable	Deaths (person-years)	Mortality rate (per 1,000 person-years)	Crude rate ratio (95% CI)	Adjusted rate ratio‡ (95% CI)
Current age (years)			***P*=0.001**†	***P*=0.16**†
14–29	2 (446)	4.5	0.27 (0.06–1.27)	**0.41 (0.09–1.96)**
30–39	8 (480)	16.7	1.00	**1.00**
40–49	9 (231)	39.0	2.34 (0.90–6.05)	**1.81 (0.69–4.72)**
≥50	8 (171)	46.8	2.81 (1.05–7.48)	**1.61 (0.58–4.50)**
Sex			***P*=0.23**†	***P*=0.85**†
Female	11 (693)	15.9	1.00	1.00
Male	16 (635)	25.2	1.59 (0.74–3.42)	1.08 (0.48–2.45)
Marital status*			***P*=0.10**†	***P*=0.09**†
Married	13 (885)	14.7	1.00	1.00
Single	2 (120)	16.7	1.14 (0.26–5.04)	1.18 (0.26–5.49)
Widowed/Divorced	11 (307)	35.8	2.44 (1.09–5.44)	3.21 (0.64–16.16)
Alcohol consumption			***P*=0.37**†	***P*=0.11**†
Yes	18 (986)	18.3	1.00	1.00
No	9 (340)	26.4	1.45 (0.65–3.22)	2.07 (0.87–4.96)
Tobacco use*			***P*=0.08**†	***P*=0.90**†
No	11 (884)	12.4	1.00	1.00
Yes	11 (417)	26.4	2.12 (0.92–4.89)	0.94 (0.37–2.40)
Body mass index (Kg/m^2^)*			***P*<0.001**†	***P*<0.001**†
≥18.5	8 (1,101)	7.3	1.00	**1.00**
17.5–<18.5	6 (121)	49.8	6.85 (2.38–19.73)	**4.52 (1.54–13.32)**
<17.5	12 (97)	124.3	17.10 (6.99–41.84)	**6.11 (2.30–16.20)**
Haemoglobin (g/dL)*			***P*=0.005**†	***P*=0.09**†
≥11.0	17 (1,100)	15.8	1.00	1.00
<11	8 (134)	59.7	3.78 (1.63–8.76)	0.43 (0.17–1.07)
CD4 category			***P*=0.23**†	***P*=0.65**†
≥500 cells/mm^3^	15 (884)	17.0	1.00	1.00
350–499 cells/mm^3^	12 (443)	27.1	1.60 (0.75–3.41)	1.22 (0.56–2.67)
WHO stage			***P*<0.001**†	***P*<0.001**†
1	8 (987)	8.1	1.00	**1.00**
2	6 (241)	24.9	3.07 (1.07–8.85)	**2.36 (0.77–7.19)**
3 and 4	13 (89)	145.5	17.94 (7.44–43.29)	**10.18 (3.82–27.15)**
Malaria during follow-up*			***P*=0.44**†	***P*=0.81**†
No	23 (1,151)	20.0	1.00	1.00
Yes	2 (169)	11.8	0.59 (0.14–2.51)	0.84 (0.20–3.61)
Diarrhoea>1 month			***P*=0.18**†	***P*=0.48**†
No	26 (1,319)	19.7	1.00	1.00
Yes	1 (9)	112.1	5.69 (0.77–41.90)	2.26 (0.29–17.41)
Viral load (copies/ml)*			***P*=0.03**†	***P*=0.45**†
<25,000	5 (467)	10.7	1.00	1.00
25,000–<100,000	4 (268)	14.9	1.39 (0.37–5.20)	0.92 (0.24–3.47)
100,000–<250,000	6 (114)	52.5	4.90 (1.49–16.05)	2.37 (0.69–8.17)
≥250,000	3 (64)	47.2	4.41 (1.05–18.45)	1.67 (0.35–7.00)
Calendar period			***P*=0.67**†	***P*=0.27**†
1992–1999	5 (381)	13.1	1.00	1.00
2000–2003	7 (315)	12.2	1.69 (0.54–5.33)	1.60 (0.50–5.11)
2004–2007	8 (350)	22.8	1.74 (0.57–5.32)	1.82 (0.57–5.78)
2008–2011	7 (281)	24.9	1.90 (0.60–6.00)	3.22 (0.99–10.39)

* Variables with some missing data. For all variables except haemoglobin and viral load, data are missing for <2% of total observation time. Haemoglobin missing for 8.8% of observation time (117 PY) and viral load missing for 31.3% (415 PY).

† *P*-value from likelihood ratio test.

‡ Adjusted for age (a priori) and all independent predictors of mortality (variables in bold): age, BMI and WHO stage. The final model was based on 1,308 PY of observation time and 26 deaths.

## Discussion

In this study, we found that mortality among individuals with CD4 cell count 350–499 cells/mm^3^ was higher than that among people with CD4 ≥500 cells/mm^3^, although this was not statistically significant. Individuals with CD4 cell count 350–499 cells/mm^3^ and ≥500 cells/mm^3^ had 146% and 123% higher risk of death compared with the general population of Uganda. After adjusting for age, increasing WHO clinical stage and decreasing BMI were independent predictors of death.

Among the strengths of this study was the prospective study design, which allowed measurement of the main exposure of interest (CD4 cell count), and other time-varying exposures. There was active tracing of participants who missed their scheduled visits which ensured good compliance, minimized loss to follow-up and reduced number of missed deaths. Participant recruitment was done from a general population source which minimized selection bias and made study findings more representative of the population. The long study duration enabled us to accrue a reasonable number of person-years of observation.

However, the study limitations included the small numbers of deaths which makes mortality estimates from our study subject to substantial reduction in statistical power. The study lacked information on the duration of HIV infection for participants who had been recruited as prevalent cases. Since disease duration is a key factor known to influence mortality among HIV-infected individuals, survival bias could not be excluded. The analysis did not take into account of the confounding effect of tuberculosis which is prevalent in the study area and a major cause of death. Some of the time-varying variables such as smoking and education were not recorded during follow-up. The big proportion of observation time in which some exposure variables such as CD4 cell count (the main exposure of interest) and viral load were missing had an impact statistical power. Lastly, there was no adjustment made for cotrimoxazole prophylaxis on the individual level since these data were not available; however, we did adjust for calendar period which would have captured some of this effect, since cotrimoxazole was prescribed for all patients after 2004.

Although not significant due to small numbers, the higher mortality among individuals with CD4 cell count 350–499 cells/mm^3^ compared with CD4 cell count ≥500 cells/mm^3^ is an indication of the increased risk of mortality associated with deteriorating immune status which has been reported in previous studies (30, 31). Our reported MRs in the CD4 categories of 350–499 and ≥500 cells/mm^3^ (17.0 and 27.1 per 1,000 PY, respectively) are similar to what has been reported in a pre-ART follow-up study done in Ivory Coast (32). However, our observed MRs are lower than what was reported in a pre-ART study in West Africa (33). The latter was a bigger study based on data from five longitudinal cohorts. However, that study did not have data from a reference population to enable the calculation of SMRs to compare observed mortality with background population mortality.

Individuals who are on ART and have achieved high CD4 cell count have been reported to have a similar mortality experience to that of the general population (34). Therefore, one would expect a similar mortality experience among HIV-infected ART naïve individuals with comparable immune status. However, our results indicate that people with CD4 cell count ≥350 cells/mm^3^ have a higher risk of death than that in the general population of Uganda. A study in France also reported a higher overall mortality among individuals on combinational ART than in the general population. However, among individuals who had attained CD4 cell counts ≥500 cells/mm^3^, the mortality which was higher than that in the general population up to 3 years on ART reached the level in the general population after the sixth year on ART (35). In reality, not offering ART to people with CD4 cell count ≥350 cells/mm^3^ is mainly due to cost implications and concerns about long-term potential side effects, but it may also indirectly imply that the risk of death among this group of individuals is low. In our cohort, where participants were regularly reviewed by study clinicians and received free medical care, one would expect mortality to be similar to that seen in the general population of Uganda. There is evidence that participants in cohorts with free access to investigations and treatment are less likely to die (32) and therefore mortality estimates from our cohort are likely to underestimate mortality of HIV-positive individuals in Uganda in general.

Our results raise three important questions for physicians and policy makers in the developing countries where the threshold for starting ART is ≤350 cells per mm^3^: 1) Are we still waiting too long to start HIV-infected individuals on ART? 2) Are we actually treating all individuals who need ART? 3) Is it still necessary to start people on ART basing on CD4 cell count cut-offs? The above questions have to be answered in the context of available evidence and the economical implications of starting ART at higher CD4 cell counts in the developing world.

Although this study is based on small numbers, it supplements the existing evidence that mortality among HIV-infected people with high CD4 cell count is higher than that in the general population. More light will be shed from results of an ongoing multi-site randomized START Trial that is assessing whether immediate initiation of ART at CD4 cell counts above 500 cells/mm^3^ is superior to deferral of ART until CD4 cell count declines below 350 cells/mm^3^ (36). Nevertheless, long-term benefits of ART, especially in reducing HIV-associated mortality and morbidity and reducing HIV transmission, clearly outweigh the cost implications which are often cited in sub-Saharan Africa.

Increasing WHO clinical stage and decreasing BMI were identified as predictors of death among individuals with CD4 cell count above ≥350 cells/mm^3^. WHO clinical stage and BMI could both reflect underlying morbidity such as tuberculosis which is common among HIV-infected persons in sub-Saharan Africa and is a major cause of death. Low BMI can also reflect other conditions like malnutrition and HIV wasting syndrome which are associated with HIV infection and are well known to lead to poor survival. These findings further confirm the importance of WHO clinical stage assessment in HIV care, especially in this era when CD4 cell count is increasingly becoming available to clinicians.

## Conclusion

This study provides additional evidence that mortality among HIV-infected ART naïve individuals with CD4 cell count ≥350 cells per mm^3^ is higher than that in the general population. To avert these preventable deaths, ART guidelines in developing countries should aim to initiate ART at higher CD4 cell counts than the current threshold, as is done in developed countries. Consequently, there is a need to allocate more resources for the expected increase in patients initiating ART at the higher CD4 threshold. After adjusting for current age, WHO clinical stage and BMI were the main predictors of mortality, and they should be considered during the clinical assessment for ART eligibility.
